# The Role of Oxidative Stress in the Longevity and Insecticide Resistance Phenotype of the Major Malaria Vectors *Anopheles arabiensis* and *Anopheles funestus*

**DOI:** 10.1371/journal.pone.0151049

**Published:** 2016-03-10

**Authors:** Shüné V. Oliver, Basil D. Brooke

**Affiliations:** 1 Vector Control Reference Laboratory, Centre for Opportunistic, Tropical and Hospital Infections, National Institute for Communicable Diseases, Sandringham, Johannesburg, South Africa; 2 Wits Research Institute for Malaria, School of Pathology, Faculty of Health Sciences, University of the Witwatersrand, Parktown, Johannesburg, South Africa; Instituto Nacional de Salud Pública, MEXICO

## Abstract

Oxidative stress plays numerous biological roles, both functional and pathological. The role of oxidative stress in various epidemiologically relevant biological traits in *Anopheles* mosquitoes is not well established. In this study, the effects of oxidative stress on the longevity and insecticide resistance phenotype in the major malaria vector species *An*. *arabiensis* and *An*. *funestus* were examined. Responses to dietary copper sulphate and hydrogen peroxide were used as proxies for the oxidative stress phenotype by determining the effect of copper on longevity and hydrogen peroxide lethal dose. Glutathione peroxidase and catalase activities were determined colorimetrically. Oxidative burden was quantified as protein carbonyl content. Changes in insecticide resistance phenotype were monitored by WHO bioassay. Insecticide resistant individuals showed an increased capacity for coping with oxidative stress, mediated by increased glutathione peroxidase and catalase activity. This effect was observed in both species, as well as in laboratory strains and F_1_ individuals derived from wild-caught *An*. *funestus* mothers. Phenotypic capacity for coping with oxidative stress was greatest in strains with elevated Cytochrome P450 activity. Synergism of oxidative stress defence enzymes by dietary supplementation with haematin, 3-Amino-1, 2, 4-triazole and Sodium diethyldithiocarbamate significantly increased pyrethroid-induced mortality in *An*. *arabiensis* and *An*. *funestus*. It is therefore concluded that defence against oxidative stress underlies the augmentation of the insecticide resistance phenotype associated with multiple blood-feeding. This is because multiple blood-feeding ultimately leads to a reduction of oxidative stress in insecticide resistant females, and also reduces the oxidative burden induced by DDT and pyrethroids, by inducing increased glutathione peroxidase activity. This study highlights the importance of oxidative stress in the longevity and insecticide resistance phenotype in malaria vectors.

## Introduction

An inevitable consequence of aerobic physiology and metabolism is oxidative stress. This particular form of metabolism results in the formation of unstable and reactive intermediates of oxygen known as reactive oxygen species (ROS). ROS are needed for several crucial biological functions, but excessive amounts of these molecules result in a state known as oxidative stress [1]. Oxidative stress underlies numerous pathological conditions and is also a focus of biogerontology as it has been identified as an underlying accelerant of the ageing process [2]. Flying and haematophagous insects are subject to some of the greatest levels of oxidative stress in the animal kingdom [3]. In haematophagous insects including mosquitoes, blood ingestion induces oxidative stress as a consequence of the release of iron during the digestion of haemoglobin [4].

*Anopheles arabiensis* and *An*. *funestus* are two of Africa’s dominant malaria vector species (DVS) and are the predominant vectors in southern Africa [5]. Although these species differ in their feeding and resting behaviours they are often found in sympatry. *Anopheles funestus* is highly endophilic and anthropophilic, and tends to breed in semi-permanent bodies of water. *Anopheles arabiensis*, a member of the *An*. *gambiae* complex, is expophilic and endophilic as well as anthropophilic and zoophilic, and tends to breed in clear, temporary bodies of water [5] *Anopheles funestus* is the most efficient vector of malaria in Africa [6], while *An*. *arabiensis* is often implicated in lower-level residual malaria transmission owing to its behavioural plasticity [7,8]. Therefore, these vector species present different but important challenges to vector control efforts.

Adult vector longevity and insecticide resistance are especially important phenotypes in terms of malaria epidemiology and the latter can adversely affect vector control interventions if not appropriately managed [9,10,11]. The longer a female vector lives, the more likely she is to reach and exceed the intrinsic incubation period of the *Plasmodium* parasite. Therefore, small changes in longevity can have a significant effect on vector competency [11]. As malaria vector control is primarily based on the use of insecticides, insecticide resistance can diminish the efficacy of this approach ultimately leading to control failure [12]. Oxidative stress and responses to oxidative damage can inadvertently affect both of these traits.

The first reported study of the molecular basis of longevity in a malaria vector species demonstrated a link between oxidative stress and longevity [13]. This study suggested that insecticide resistant and susceptible individuals have differing capacities for coping with oxidative stress. Specifically, it was suggested that reduced longevity in insecticide resistant individuals is a direct consequence of increased oxidative stress. An unrelated study in the major malaria vector *An*. *arabiensis* also highlighted the importance of oxidative stress in vector mosquitoes [14]. In this study multiple bloodmeals were found to augment and maintain the expression of the insecticide resistance phenotype in aging adults—well past the intrinsic incubation period for *Plasmodium* development. Furthermore, it was found that the taking of multiple bloodmeals offered a greater advantage in terms of extending the lifespan of resistant females compared to their insecticide susceptible counterparts. This study suggested that a difference in the capacity to cope with oxidative stress was the underlying mechanism behind this observation. These findings were built on a previous study which showed that Glutathione S-transferases (GSTs) with peroxidase activity were involved in protection against pyrethroid intoxication in the brown plant hopper *Nilaparvata lugens* [15]. Gravid pyrethroid resistant *An*. *arabiensis* females from Northern Cameroon had elevated GST and oxidative stress enzyme activity, but generally suppressed Cytochrome P450 activity [16]. This highlights the interplay between insecticide resistance, particularly pyrethroid resistance, and oxidative stress.

As oxidative stress plays an important role in mediating longevity and the expression of insecticide resistance, the aim of this study was to examine the role of defence against oxidative stress on these phenotypes in *An*. *arabiensis* and *An*. *funestus*. Furthermore, this study also aimed to examine whether defence against oxidative stress is the mechanism that underlies the augmentation of the resistance phenotype by multiple blood-feeding in *An*. *arabiensis*.

## Materials and Methods

### Mosquito strains

All strains used in this study were maintained in the Botha de Meillon insectary, NICD, Johannesburg, under standard culturing conditions of 25°C (±2°C) and 85% humidity (±5%) [17].

#### Anopheles arabiensis

SENN: Colonised from material collected in Sennar, Sudan, in 1980. This strain shows low levels of pyrethroid resistance, but is otherwise fully susceptible to all other insecticide classes.

SENN DDT: Selected for resistance to DDT from the SENN strain in 1995. This strain regularly undergoes DDT selection, and displays resistance to DDT, permethrin, deltamethrin, malathion [14] and λ-cyhalothrin (data not shown). Resistance is mediated by a combination of elevated GST, Cytochrome P450 and general esterase activity and this strain is also fixed for the L1014F *kdr* mutation[18].

#### Anopheles funestus

FANG: Colonised from material collected from Calueque, southern Angola in 2002. This strain is fully susceptible to all insecticides.

FUMOZ: Colonised from material collected in southern Mozambique in 2000. This strain has not been selected for resistance, but retains appreciable levels of pyrethroid and carbamate resistance.

FUMOZ-R: Selected for resistance to permethrin from the FUMOZ strain. This strain retains comparatively high levels of resistance to pyrethroids and carbamates, but is not currently undergoing selection.

Clade I: Pooled F1 progeny obtained from wild-caught *An*. *funestus* females caught in Nchelenge, Zambia, in January 2014. Each female (mother) was identified as *An*. *funestus* Clade I according to a previously described method [19]. The wild population from which these progeny were derived showed resistance to deltamethrin and bendiocarb [20].

Clade II: Pooled F1 progeny obtained from wild-caught *An*. *funestus* females caught in Nchelenge, Zambia, in January 2014. Each female (mother) was identified as *An*. *funestus* Clade II according to the method described previously described [19]. The wild population from which these progeny were derived also showed resistance to deltamethrin and bendiocarb [20].

### The effect of oxidative stress on selected phenotypes

#### Oxidative stress and longevity

To assess the difference in capacity of insecticide resistant and susceptible individuals to cope with oxidative stress, copper sulphate (CuSO_4_) was used as a stressing agent. CuSO_4_ is commonly used in agriculture [21,22] and its’ toxic effect is due to the induction of oxidative stress [23]. The SENN and SENN DDT strains were used in this experiment. A 10% sucrose solution supplemented with CuSO_4_ solution to a final concentration of 10mM was placed in each of three cages each containing 25 newly emerged females. This solution was the only source of carbohydrate available in the treatment cages. Age-matched cages in which only un-supplemented 10% sucrose was available served as controls. None of the females were allowed to mate and they were not offered any bloodmeals during the course of their lifetime. Sucrose solutions were changed every second day, and mortality was assessed daily, with cadavers removed on the day of death. Mortality was monitored until all individuals were dead. This experiment was replicated 3 times, each time using females that emerged from a separate egg batch. Mortality was assessed using the Kaplan-Meier estimator, with the log rank test used to compare survival in the treatment versus control cages.

#### Oxidative stress and insecticide resistance

To determine which factors could drive differential responses to oxidative stress in insecticide resistance, the relationship between insecticide exposure and oxidative stress was examined at phenotypic and biochemical levels. Two hundred 3–5 day old non-bloodfed SENN DDT females were exposed to either 4% DDT, 0.75% permethrin, 0.05% deltamethrin or 5% malathion by WHO bioassay [24]. Adults that survived 24 hours post exposure were maintained for longevity analysis. These females were maintained unmated and without blood on a diet of 10% sucrose for the remainder of their lives. Age matched, insecticide unexposed females served as controls. Cadavers were removed daily, and mortality was monitored until all individuals were dead. This experiment was replicated 3 times, each time using females that emerged from a separate egg batch. Mortality was assessed using the Kaplan-Meier estimator which was used to compare survival between insecticide treatments and between treatments and controls. The log rank test was used to determine whether any differences in longevity were significant.

To determine the biochemical relationship between insecticide exposure and oxidative stress the protein carbonyl content of insecticide exposure survivors was determined. Three hundred 3–5 day old non-bloodfed SENN DDT females were exposed to either DDT, permethrin, deltamethrin or malathion by WHO bioassay [24]. Adults that survived 24 hours post exposure were collected and immediately frozen at -70°C. Age matched unexposed females were used as comparative controls. Protein carbonyl content was determined using the DNPH derivitivization method [25] modified for insects as described in Vontas *et al*., [15] and Oliver and Brooke [14]. The mean protein carbonyl contents of the treated samples were compared to those of the controls using 1-way ANOVA.

#### The effect of dietary hydrogen peroxide on mortality

The capacity to cope with hydrogen peroxide consumption is a common method used to measure the capacity of *D*. *melanogaster* to cope with oxidative stress [26]. In this study, three factors were examined. The first was to determine whether male and female mosquitoes differ in their capacity to cope with oxidative stress. The second was to determine whether there was a difference between strains selected for resistance and their respective baseline strains. Thirdly, the effect of metabolic resistance only (FUMOZ-R) was compared to resistance mediated by both metabolic resistance and *kdr* (SENN-DDT) by comparing the capacity to cope with oxidative stress between FUMOZ-R and SENN-DDT.

The LD_15_ for hydrogen peroxide of male and female SENN and SENN DDT as well as FUMOZ and FUMOZ-R was determined using a concentration range of 0, 5, 10, 15, 20 and 25% hydrogen peroxide solution prepared in 10% sucrose. Pure hydrogen peroxide (30%) was not used as the mosquitoes avoided these solutions. Exposure assays were set up in which a container with a single concentration of sugar/hydrogen peroxide solution was introduced into a cage of 75 individuals, either male or female, of each strain. Three-day-old non-bloodfed individuals starved of sugar for 12 hours were used. Mortality at each concentration was assessed within 24 hours of supplying the sugar/hydrogen peroxide solution. Lethal Doses calculated from 24 hour post exposure mortalities were compared (males vs females by strain, FUMOZ-R vs FANG by gender, SENN-DDT vs SENN by gender, FUMOZ-R vs SENN-DDT by gender) using a 1-way ANOVA.

#### Comparisons of oxidative stress defence enzyme activity

Based on the findings of the phenotypic studies, the enzyme activities of catalase and glutathione peroxidase were assessed colorimetrically as a biochemical measure of oxidative stress defence. The activity of each strain was determined, but the oxidative stress enzyme experiment pertaining to *An*. *arabiensis* is discussed in the context of multiple blood-feeding.

Glutathione peroxidase activity was determined as a measure of Nicotinamide adenine dinucleotide phosphate (NADPH) consumption [15]. Catalase activity was based on the hydrogen peroxide consumption assay [27]. It was adapted for microplate assay by scaling down the reaction to 200μl [14]. Samples were prepared by homogenising single 5–7 day-old non blood-fed, non insecticide-exposed adults in 50mM 0.1M Sodium phosphate pH7.0, with all assays being performed within 48 hours of homogenisation. The crude homogenate served as a protein source for both assays. Protein was quantified using the Bradford method, with a standard curve calibrated on Bovine Serum Albumen (BSA) [28]. For all experiments, two blank readings were included. This constituted of a complete reaction mixture without protein homogenate. This ensured that any changes in optical density were due to the addition of protein. Ninety six laboratory reared mosquitoes (FANG, FUMOZ, FUMOZ-R) per gender and strain were assayed while 48 F1 Clade I and Clade II mosquitoes were assayed per gender and strain. The enzyme activity of the individuals was compared between strains and by gender using 1-way ANOVA, with a Tukey HSD as a post hoc test.

#### Synergism of oxidative stress enzymes

Exposure assays using synergist compounds are a common method of determining which detoxification enzymes are involved in the production of an insecticide resistance phenotype [29,30]. The effect of synergists on oxidative stress enzymes in *Drosophila melanogaster* was examined by introducing the synergist into their diet [23]. Based on this study, synergists were introduced to SENN DDT and FUMOZ-R as a sucrose supplement. The Copper-Zinc Superoxide dismutase synergist 3-Amino-1, 2, 4-triazole (ATZ) was provided as a 17mM solution, the Catalase synergist Sodium diethyldithiocarbamate (DDC) was provided as a 2mM solution and the Glutathione S-transferase (GST) synergist haematin was provided as a 3.6x10^-4^ mM solution. The synergist/sucrose solutions were provided to 48 hour old non blood-fed adults which were starved for 12 hours prior to the provision of the solutions. These adults were allowed to feed on the solution for 24 hours, and the 3-day-old individuals were then used for standard WHO bioassays [24].

The optimal synergist concentration was determined as follows: Two cages containing equal numbers of freshly emerged adults were prepared. Each cage received either a standard sugar solution or a synergist treated sugar solution. The adults were allowed to feed on the sugar solution for three days, as it was assumed that any adult that did not imbibe the solution would not survive. The highest soluble synergist concentration found not to induce a significant increase in mortality compared to the control was selected.

After the synergist treatment, the adults were exposed in the standard WHO bioassay. Mortality was scored 24 hours post exposure. Two sets of controls were put in place. Unexposed control mortality (mosquitoes not exposed to synergist) constituted an environmental control. Mosquitoes exposed to synergist but not insecticide constituted a second control. Assays were accepted if both sets of controls had a 24 hour mortality of below 5%. Results were analysed using 1-way ANOVA.

#### The interaction between multiple bloodmeals and oxidative stress in *An*. *arabiensis*

**Oxidative stress variation with bloodfeeding and insecticide exposure:** As a baseline role for the defence against oxidative stress in the insecticide resistance phenotype was previously established, the following experiments aimed to establish whether oxidative stress plays a role in the maintenance of insecticide resistance expression with age as a consequence of multiple blood-feeding as previously assessed [14].

As blood is a complex, protein-rich substance, it is an inhibitor of various biochemical assays, and also affects the optical density readings of spectrophotometric assays [31]. As such, the following procedure was used to avoid blood contamination in the assays. After the final blood treatment, females were allowed to digest the meal for 72 hours in order to allow the blood to be fully metabolised [32]. The females were then killed and assessed for oxidative stress. Note that the samples were named after the day they received their last blood treatment as opposed to the day they were killed. Mosquitoes were blood-fed by a single, consenting human volunteer. This was performed according to the guidelines outlined in the ethical clearance certificate obtained by the Faculty of Health Sciences ethics committee of the University of the Witwatersrand (Clearance number M130534), and the single volunteer consented in writing.

Protein carbonyl content is a common measure of oxidative stress, and carbonyl content was determined by the 2,4-Dinitrophenylhydrazine derivitivization method [25], as modified by Vontas *et al*., [15]. For the first experiment unexposed SENN and SENN DDT individuals were used. For each strain 0.15g (approximately 150 individuals) was used as starting material. For each strain, one of three treatments corresponding to age was prepared. The first was a sugar-fed control. The second treatment was a group that had received a single bloodmeal at the age of 15 days. The third treatment was a group that had received bloodmeals at the ages of 3,7, 11 and 15 days of age. These groups were named 0 blood, 1 blood and multiblood respectively, and killed as described earlier. All experiments were replicated 3 times using individuals derived from separate egg batches. Protein carbonyl content was compared between treatments using 1-way ANOVA.

For the second experiment, only SENN DDT females were used. The following treatments were prepared: 15 day sugar-fed, unexposed (denoted sugar), 15 day sugar fed and exposed to DDT (denoted DDT), unexposed but fed a single bloodmeal at age 15 days (denoted single blood), exposed to DDT after being fed a single bloodmeal at day 15 (denoted single blood+DDT), unexposed but fed bloodmeals at ages 3, 7, 11 and 15 days (denoted multiblood) and finally a group exposed to DDT at age 15 days after receiving a bloodmeal at ages 3, 7, 11 and 15 days (denoted multiblood+DDT). Samples were prepared as previously described. All experiments were replicated 3 times using individuals derived from separate egg batches. Protein carbonyl content was compared between treatments using 1-way ANOVA.

## Results

### The effect of oxidative stress on longevity

**Fig 1:**
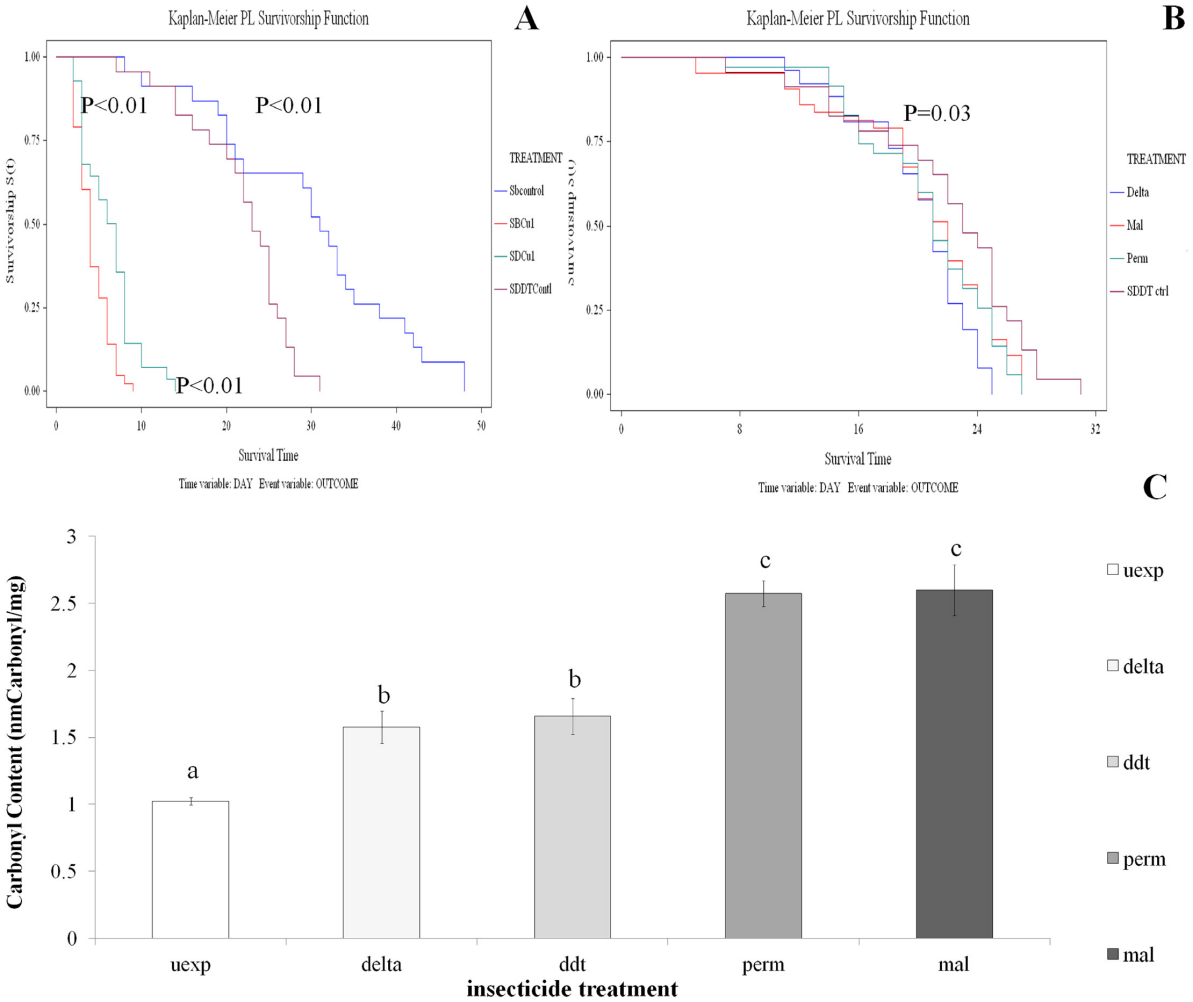
Longevity and oxidative stress in *An*. *arabiensis*. A: The effects of copper sulphate exposure on longevity. Under unstressed conditions SENN females (blue) lived significantly longer than SENN DDT (purple) females. Copper sulphate exposure significantly reduced longevity in both strains, but under conditions of oxidative stress SENN DDT females (green) lived significantly longer than SENN females (red). B: The effect of insecticide exposure on subsequent longevity of SENN DDT. Survivors following insecticide exposures (deltamethrin, malathion, permethrin) lived for a significantly shorter period than their unexposed counterparts (purple). C: The effects of insecticide exposure on the oxidative burden of SENN DDT females. All insecticides significantly increase protein carbonyl content. Deltamethrin and DDT induced the same level of carbonyl content, while malathion and permethrin induced the same level of carbonyl content.

The effect of oxidative stress on longevity was assessed. The first method examined the response of insecticide resistant and susceptible strains to the common dietary stressor copper sulphate. The SENN and SENN DDT strains responded differently to dietary induced oxidative stress, although under non-stressed conditions, the unselected SENN strain lived significantly longer than their selected SENN DDT counterparts (Log rank test p<0.01, χ2 = 9.66). Although copper sulphate supplementation significantly reduced longevity in both strains (Log rank test p<0.01; χ2 = 75.31), the selected SENN DDT strain samples lived significantly longer than their unselected SENN counterparts (Log rank test p<0.01; χ2 = 10.95) (Fig 1A).

As the insecticide resistant SENN DDT strain coped better with oxidative stress than their susceptible SENN counterparts, it was decided to examine the effect of insecticide-induced oxidative stress on exposure survivors. When examining the longevity of insecticide exposure survivors, it was observed that, with the exception of DDT exposure, insecticide exposure always significantly reduced the longevity of survivors (Log rank test p = 0.03; χ2 = 8.62) (Fig 1B). In terms of the oxidative burden induced by insecticide exposure, all four insecticides induced a significant increase in protein carbonyl content compared to the control (1-way ANOVA: p<0.01; df = 4; F = 30.0) (Fig 1C).

### Variation in oxidative stress defence phenotype in insecticide selected and unselected strains

As insecticide resistant *An*. *arabiensis* were found to be more tolerant of oxidative stress, it was decided to examine whether insecticide resistance mechanism played a role in the capacity to resist oxidative stress. The *An*. *funestus* strain FUMOZ R, in contrast to SENN DDT, mediates resistance by metabolic mechanisms only. Due the differences in longevity between the species, the capacity to cope with hydrogen peroxide intoxication was used as a measure of oxidative stress tolerance.

**Fig 2:**
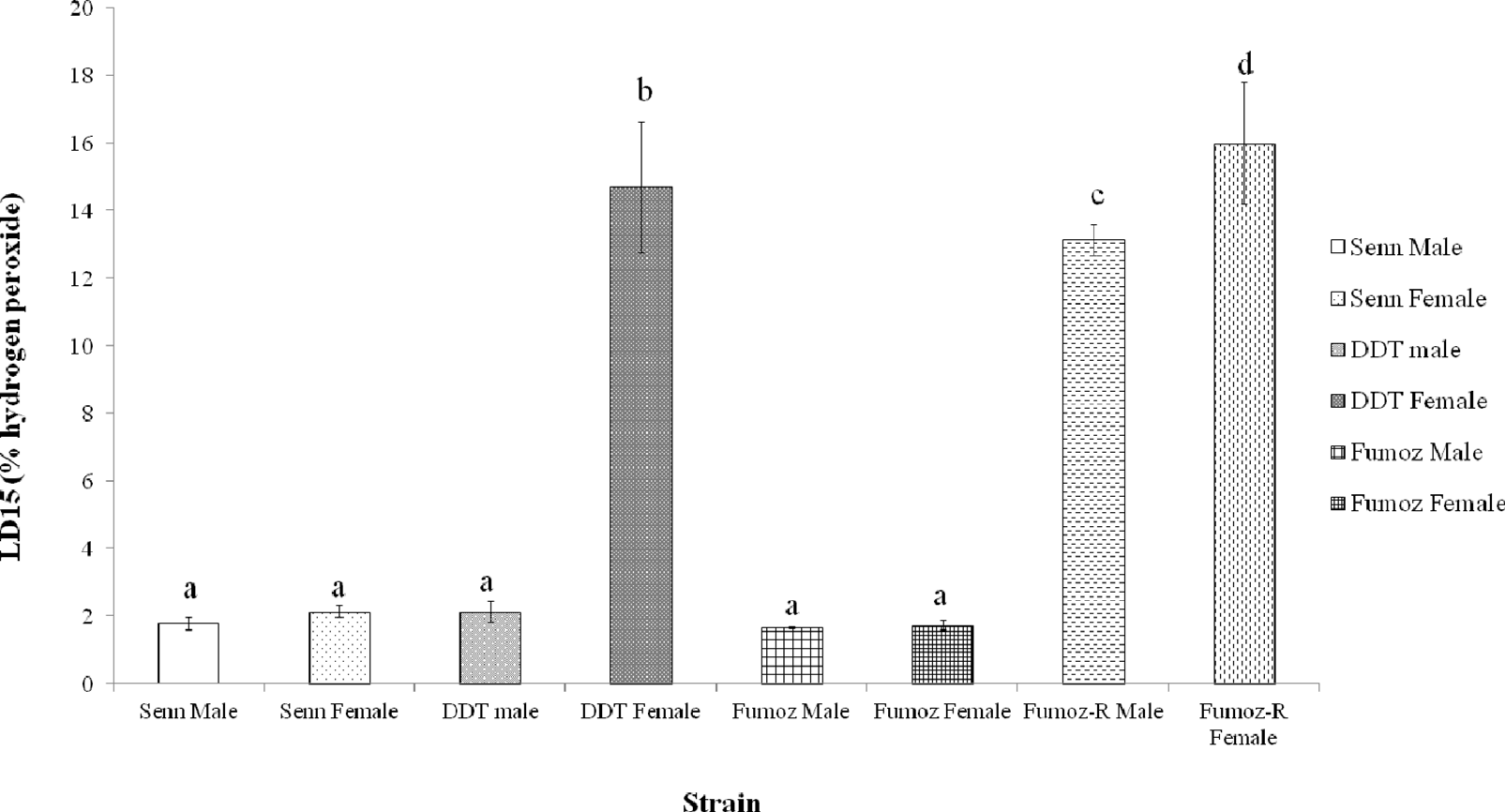
Comparison of hydrogen peroxide lethal dose inducing 15% mortality (LD_15_) in selected and unselected *An*. *arabiensis* and *An*. *funestus*. In the *An*. *arabiensis* strains only the selected SENN DDT females (DDT female) showed a significantly increased LD_15_ for hydrogen peroxide. In the *An*. *funestus* strains there was no difference in LD15 between males and females of the unselected FUMOZ strains and of the selected FUMOZ-R strain. The lethal doses for both male and female FUMOZ-R were significantly higher than their unselected counterparts. Bars denoted by the same letter indicate no significant differences in LD15. Standard errors are shown.

Fig 2 summarises the hydrogen peroxide LD_15_ values for laboratory strains of *An*. *arabiensis* and *An*. *funestus*. LD_15_ values were calculated, as LD_50_ values could not be accurately calculated for the *An*. *funestus* strains, which were highly resistant to hydrogen peroxide.

There was no significant difference in hydrogen peroxide LD_15_ between SENN males and females (2 sample t-test: p = 0.22, t = -1.47). In contrast, female SENN DDT had significantly higher LD_15_ values than their male counterparts (2 sample t-test: p = 0.01; t = -6.43). SENN DDT males did not differ significantly from SENN males and females (1-way ANOVA: p = 0.38; F = 0.82).

Similar results were found in the *An*. *funestus* strains. In FUMOZ there was no significant difference between the hydrogen peroxide LD_15_ of males and females (2 sample t-test: p = 0.25, t = -1.51). FUMOZ-R females showed a significantly higher LD_15_ than FUMOZ females (2 sample t-test: p<0.01; t = 7.92). Similarly, FUMOZ-R males showed a significantly higher LD_15_ than FUMOZ males (2 sample t-test: p<0.01; t = 23.79). There was no significant difference between the LD_15_ of FUMOZ R males and females (2 sample t-test: p = 0.25; t = 1.54) or between male and female FUMOZ (2 sample t-test: p = 0.68; t = 0.45). There was also no significant difference in LD_15_ between male SENN DDT and FUMOZ and SENN adults of both genders (1-way ANOVA: p = 0.30; F = 1.40; df = 4).

### The biochemical basis of oxidative stress resistance

The previous experiments demonstrated that insecticide resistant mosquitoes had a better oxidative stress resistance capacity than their susceptible counterparts. The following sets of experiments aimed to determine the biochemical basis of these observations.

Glutathione peroxidase and catalase enzyme activity are two key mediators of the oxidative stress defence response. This experiment assayed the activity of these two enzymes in insecticide susceptible (FANG), unselected baseline resistant (FUMOZ) and selected insecticide resistant (FUMOZ R) *An*. *funestus* strains. The activity of these enzymes in *An*. *arabiensis* stains are described in a later set of experiments.

**Fig 3:**
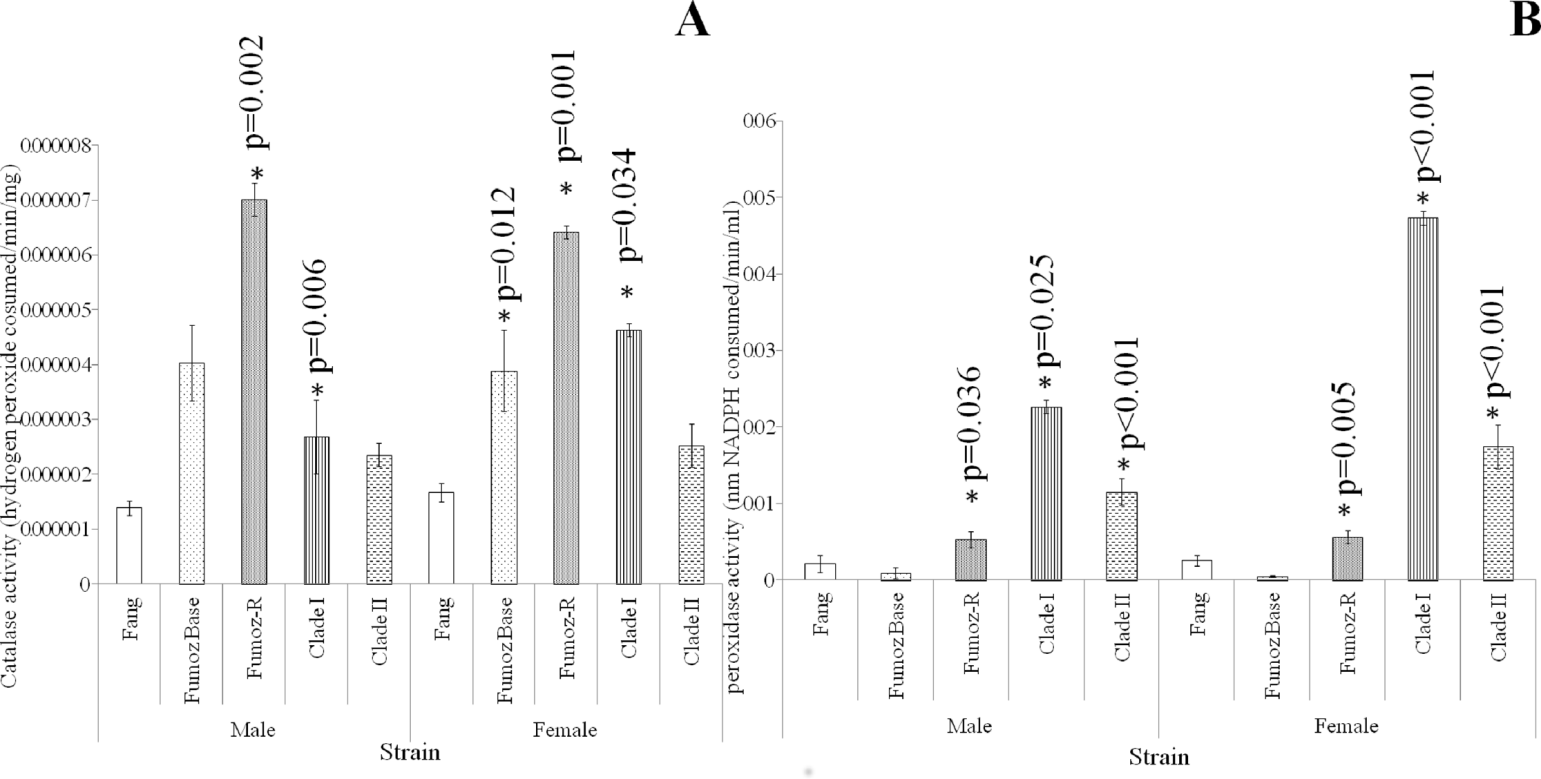
Comparative activities of oxidative stress enzymes (catalase and peroxidase) in wild and laboratory reared *An*. *funestus* strains. A: FUMOZ-R males and females showed significantly higher catalase activity than their susceptible and unselected counterparts (FANG and FUMOZ BASE). FUMOZ BASE females showed higher catalase activites than their susceptible counterparts, while males did not. Clade I females and males showed significantly higher activities than their FANG counterparts. Clade II samples did not differ significantly from FANG in catalase activities. B: FUMOZ-R males and females showed significantly higher Glutathione peroxidase activities than their FANG and FUMOZ counterparts. The peroxidase activities of Clade I and II males and females are significantly higher than their laboratory counterparts. Clade I females showed significantly higher peroxidase activities than their Clade II counterparts. P values with an asterisk (*) indicate significant differences between the strain and FANG, while p-values with a circle (o) denote significant differences between clades.

In terms of catalase activity (Fig 3A), FUMOZ-R showed higher levels of enzyme activity compared to the susceptible FANG strain for both males (2 sample t-test: p = 0.01; t = -1.68) and females (2 sample t-test: p<0.01; t = -3.46). FUMOZ females showed significantly higher levels of catalase activity than FANG females (2 sample t-test: p = 0.01; t = -2.61). Clade I males showed significantly higher levels of catalase activity than FANG males (2 sample t-test: p = 0.01; t = -3.00) and Clade I females showed significantly higher levels of catalase activity than FANG females (2 sample t-test: p = 0.03; t = -2.12).

Glutathione peroxidase activities (Fig 3B) in FUMOZ-R were significantly higher than in FANG for males (2 sample t-test: p = 0.04; t = -2.15) and females (2 sample t-test: p = 0.01; t = -3.56). FUMOZ-R males showed significantly higher peroxidase activity than FUMOZ males (2 sample t-test: p<0.01; t = -3.56). Clade I males showed significantly higher activity than FANG males (2 sample t-test: p = 0.03; t = -2.31) and Clade II males (2 sample t-test: p<0.01; t = -4.65). Similarly, Clade I females showed significantly higher enzyme activity than FANG females (2 sample t-test: p<0.01; t = -4.88). Clade II females also showed significantly higher levels of enzyme activity than FANG females (2 sample t-test: p<0.01 t = -5.11). Clade II males showed significantly higher levels of activity than FANG males (2 sample t-test: p<0.01 t = -4.65) Unlike their male counterparts, there was a significant difference between the peroxidase activity of Clade I and Clade II females, with Clade I females showing a significantly higher level of enzyme activity (2 sample t-test: p<0.01; t = 3.12).

### Synergism of oxidative stress enzyme and the resistance phenotypes of *An*. *arabiensis* and *An*. *funestus*

**Fig 4:**
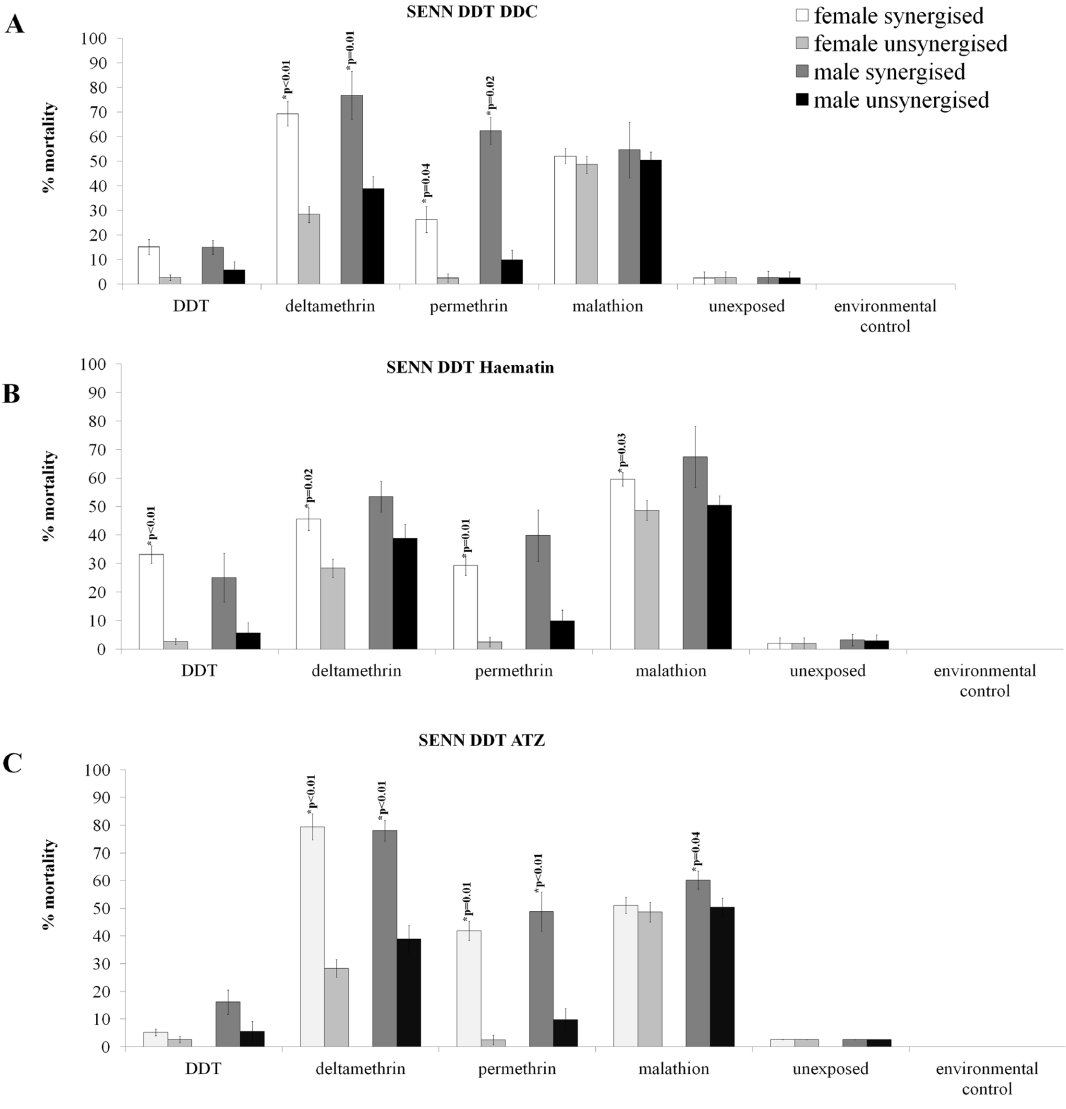
Synergism of oxidative stress enzymes and the subsequent effect on the insecticide resistance phenotype of SENN DDT. DDC synergism, which negates the activity Cu-Zn peroxidases, significantly increased the pyrethroid-induced mortality in males and females of the SENN DDT strain (A). The GST synergist haematin resulted in increased mortality induced in all classes of insecticides in female SENN DDT, but had no significant effect on insecticide induced mortality of males of the same strain (B). The catalase synergist ATZ increased pyrethroid-induced mortality of both male and female SENN DDT, as well as significantly increasing the mortality induced by malathion in SENN DDT males (C).

Fig 4A–4C shows the effect of oxidative stress enzyme synergism in *An*. *arabiensis*. In Block A, the inhibition of Cu-Zn SODs by DDC is demonstrated. Full (100%) mortality was not achieved for any of the synergised samples. Nevertheless, DDC synergism resulted in a significantly increased mortality for deltamethrin-exposed females (2 sample t-test: p<0.01; t = -6.70; n = 1199) and permethrin-exposed females (2 sample t-test: p = 0.04; t = -2.20; n = 1315). For males, DDC synergism resulted in a significantly increased mortality following deltamethrin exposure (2 sample t-test: p = 0.01; t = -2.89; n = 1210) and permethrin exposure (2 sample t-test: p = 0.02; t = -3.46; n = 1329) There was no significant difference in mortality for synergised and unsynergised males and females following malathion (♀: n = 1290, ♂: n = 1300) and DDT exposure (♀: n = 1279, ♂: n = 1272).

As with DDC synergism, haematin synergism (GST inhibition) (Fig 4B) never induced 100% mortality following insecticide exposure. Haematin synergism was the only one of three synergists that resulted in a significant increase in mortality following exposure to all four insecticides for female SENN DDT. Synergised females showed a significant increase in insecticide-induced mortality for DDT (2 sample t-test: p<0.01; t = -5.46; n = 1410), deltamethrin (2 sample t-test: p = 0.02; t = -3.40; n = 1290), permethrin (2 sample t-test: p = 0.01; t = -2.65; n = 1350) and malathion (2 sample t-test: p = 0.03; t = -2.26; n = 1310). There were no significant differences in mortality between synergised and unsynergised males (DDT: n = 1390, deltamethrin: n = 1295, permethrin: n = 1362, malathion: n = 1325).

Catalase synergism by ATZ (Fig 4C) resulted in the highest pyrethroid induced death. ATZ synergised females showed significantly increased deltamethrin-induced mortality (2 sample t-test: p<0.01; t = -8.46; n = 1375) as well as permethrin-induced mortality (2 sample t-test: p = 0.01; t = -2.85; n = 1370). Similarly, ATZ synergised males showed a significant increase in deltamethrin-induced mortality (2 sample t-test: p<0.01; t = -10.35; 1371), as well as permethrin-induced mortality (2 sample t-test: p<0.01; t = -2.09; n = 1378). Unlike the females, however, male ATZ-synergised mosquitoes showed a significant increase in malathion-induced mortality (p = 0.04; t = -3.07; n = 1211). A total of 1154 females were exposed to malathion, 1150 to DDT and 1159 males to DDT in this treatment.

The inhibition of oxidative stress enzymes in *An*. *funestus* followed a similar pattern of phenotypic change as for *An*. *arabiensis*. DDC, the SOD inhibitor, resulted in the lowest pyrethroid-induced mortality, while ATZ, the catalase inhibitor resulted in the highest insecticide-induced mortality.

**Fig 5:**
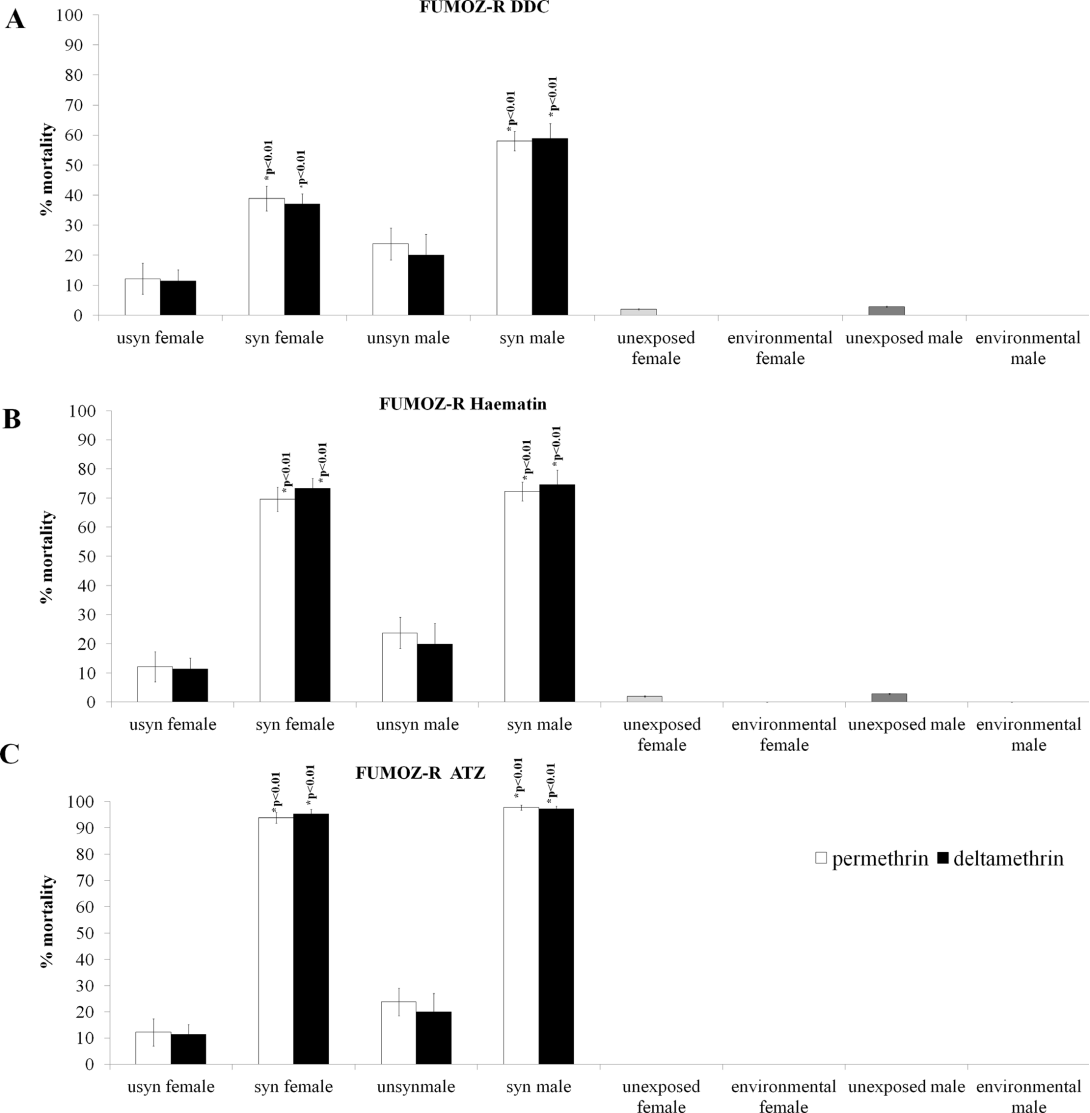
Synergism of oxidative stress enzymes and the subsequent effects on the insecticide resistance phenotype of FUMOZ R. DDC synergism significantly increased pyrethroid-induced mortality for both male and female FUMOZ-R (A). The general GST synergist haematin significantly increased pyrethroid mortality in both sexes of FUMOZ-R (B). ATZ was the most potent pyrethroid synergist of FUMOZ-R, raising pyrethroid-induced mortality to just under 100% in both males and females of the strain (C).

The SOD inhibitor DDC (Fig 5A) caused a significant increase in permethrin-induced mortality for males (2 sample t-test: p<0.01; t = -4.43; n = 1115) and females (2 sample t-test: p<0.01; t = -4.38; n = 1117). Similarly, deltamethrin-induced mortality for synergised males was significantly increased (2 sample t-test: p<0.01; t = -4.43; n = 1125) as was that of females (2 sample t-test: p<0.01; t = -3.79; n = 1100).

The GST inhibitor haematin (Fig 5B) induced higher mean pyrethroid-induced mortality than DDC. Haematin significantly increased permethrin-induced mortality for males (2 sample t-test: p<0.01; t = 7.44; n = 997) and females (2 sample t-test: p<0.01; t = -8.66; n = 989). Likewise, synergised males showed significantly increased deltamethrin-induced mortality (2 sample t-test: p<0.01; t = -6.36; n = 990) as did females (2 sample t-test: p<0.01; t = -12.9; n = 997).

The catalase inhibitor ATZ (Fig 5C) resulted in the highest overall pyrethroid induced mortality, and came the closest to inducing 100% insecticide-induced mortality. As for the other three synergists, permethrin-induced mortality was increased for males (2 sample t-test: p<0.01; t = -10.89; n = 1115) as well as females (2 sample t-test: p<0.01; t = -16.28; n = 1117). Similarly, deltamethrin-induced mortality was significantly increased for synergised males (2 sample t-test: p<0.01; t = -11.30; n = 1125) as well as synergised females (2 sample t-test: p<0.01; t = -19.68; n = 1100).

### The effect of multiple bloodmeals on the oxidative burden of *An*. *arabiensis*

**Fig 6:**
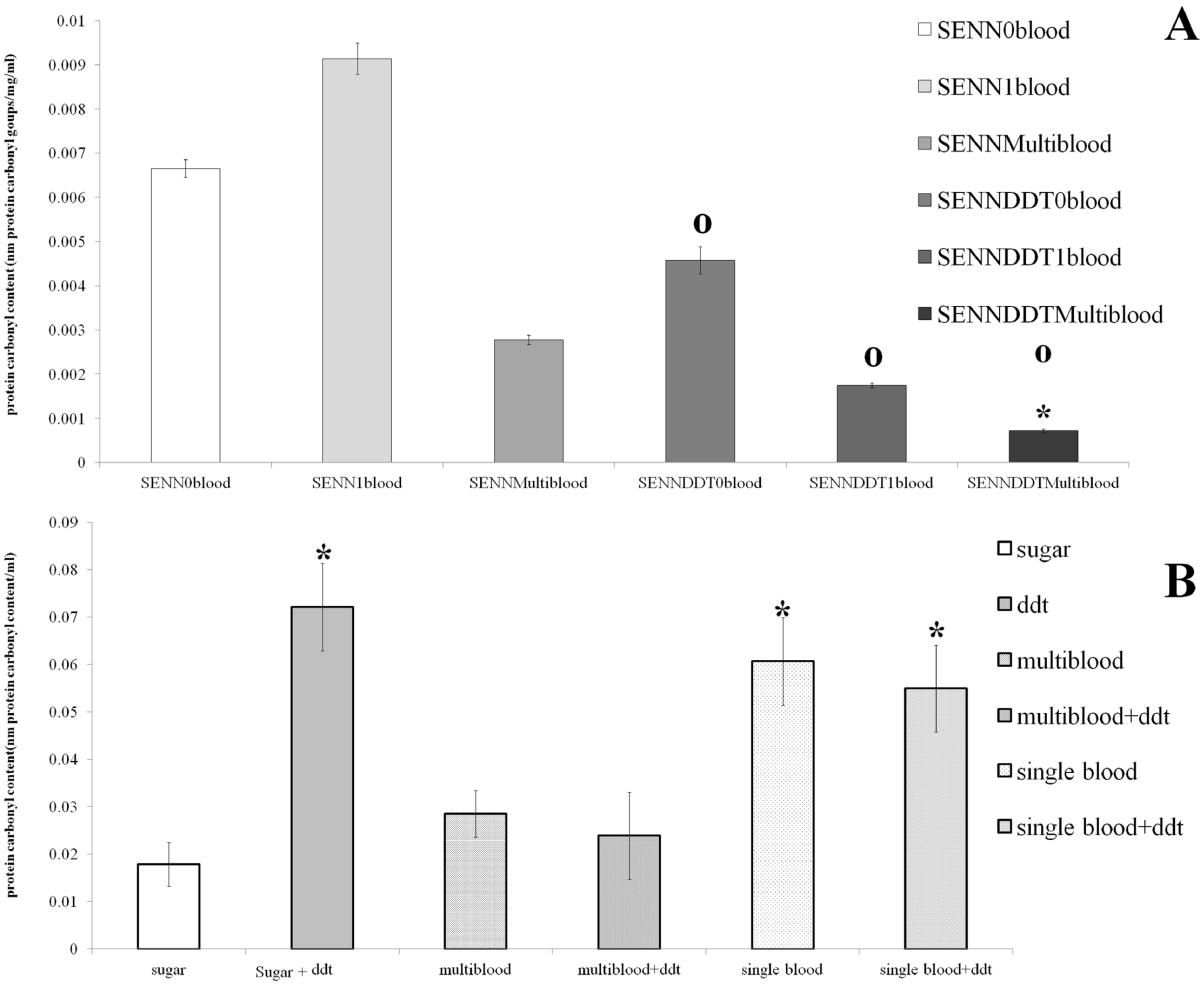
Oxidative stress measured using protein carbonyl content in *An*. *arabiensis* and its’ association with blood-feeding (single or multiple). (A) SENN DDT showed significantly lower oxidative stress than SENN, regardless of blood-feeding status. Significant differences between strains are indicated with a ‘o’ and significant differences between treatments in the same strain are indicated by an asterisk (*) (B). Exposing 3 day old non-bloodfed SENN DDT to DDT significantly increased oxidative stress. Asterisks indicate a significant increase in carbonyl content compared to unexposed, non bloodfed females (sugar). Females that had taken multiple bloodmeals prior to DDT intoxication showed significantly decreased oxidative burden after DDT exposure, but this effect was not observed in females that had only imbibed a single bloodmeal.

In Fig 6A the cumulative oxidative damage in bloodfed SENN and SENN DDT females quantified as protein carbonyl content is summarised. Multiple blood-meals resulted in a significant reduction in oxidative stress in SENN DDT females (1 way ANOVA: p = 0.02; F = 4.48, df = 1) but not SENN females (1 way ANOVA: p = 0.09; F = 2.72, df = 1). SENN DDT females had significantly lower levels of carbonyl than their SENN counterparts that had taken no blood meals (2 sample t-test; p = 0.04, t = 2.17) and multiple blood meals (2 sample t-test; p = 0.01, t = 3.31) but not those that only had a single blood meal (2 sample t-test; p = 0.12, t = 1.64).

As multiple meals had a marked effect on SENN DDT, it was decided to determine how blood-feeding interacted with insecticide-induced oxidative stress (Fig 6B). The carbonyl content of SENN DDT females that were exposed to DDT alone or in combination with blood-meals was examined. DDT exposure alone induced a significant increase in carbonyl content (2 sample t-test p = 0.01; t = -4.17), as did a single blood meal (2 sample t-test: p = 0.01; t = -2.85). A single blood meal in combination with DDT exposure also induced a significant increase in carbonyl content (2 sample t-test: p = 0.03; t = -2.45). Multiple blood meals alone induced a significantly lower oxidative burden than a single blood meal (2 sample t-test: p = 0.04; t = -2.26). Multiple meals with a DDT exposure also induced a significantly lower carbonyl content than a single meal in combination with a DDT exposure (2 sample t-test: p = 0.03; t = -2.40).

### The effect of multiple bloodmeals on the activity of oxidative stress enzymes

**Fig 7:**
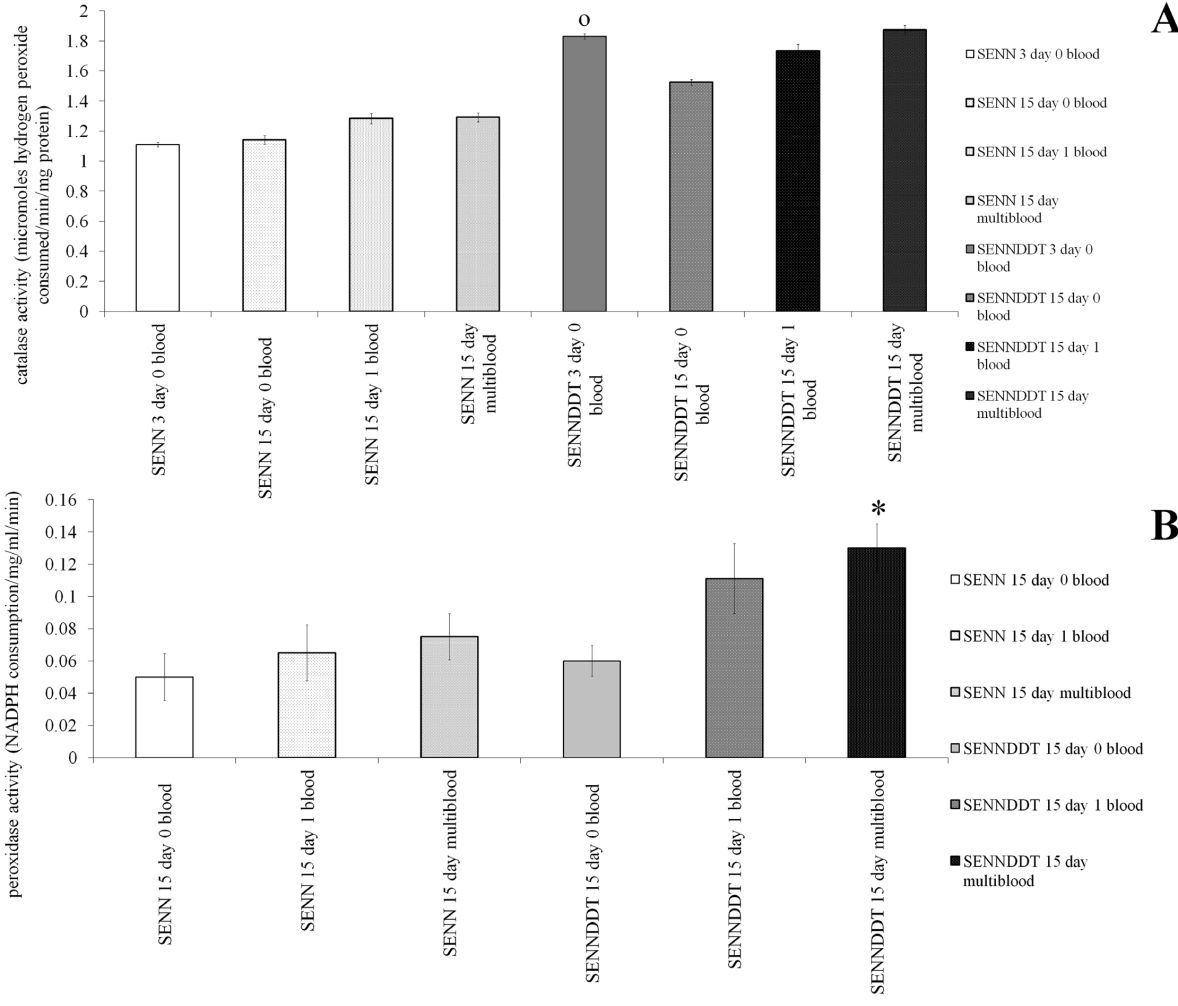
The effect of age and blood-feeding on oxidative stress enzymes (catalase and peroxidase) in *An*. *arabiensis*. (A): Blood-feeding effected the levels of catalase activity in older females, but 3-day-old SENN DDT females showed significantly higher levels of catalase activity than their equivalent SENN counterparts. (B): Blood-feeding did not affect the peroxidase activity of SENN, but multiple blood-feeding significantly increased the peroxidase activity of SENN DDT. Significant differences between strains with same treatment are denoted by a circle (O), while significant differences in bloodfeeding treatment from their unfed counterparts within the same strain are denoted with an asterisk (*).

As catalase and Glutathione peroxidase activity proved to be crucial for *An*. *funestus*, the activities of these enzymes were assayed in ageing, bloodfed female *An*. *arabiensis*. Fig 7A depicts the catalase activity of SENN and SENN DDT. Although SENN DDT females had a significantly higher level of catalase activity than their SENN counterparts (2 sample t-test: p<0.01; t = -3.04), this effect was lost later in life, where no significant differences were recorded between SENN and SENN DDT adults of 15 days of age, regardless of blood-feeding status (1-way ANOVA: p = 0.54, F = 0.82, df = 5). Blood-feeding did not result in significantly increased catalase activity in SENN (1-way ANOVA: p = 0.92, F = 0.07, df = 2) or SENN DDT (1 way ANOVA: p = 0.75, F = 0.30, df = 2).

Multiple blood-meals in SENN DDT females significantly increased the activity of Glutathione peroxidase activity (Fig 7B) compared to sugar-fed controls (2 sample t-test: p = 0.01; t = -2.45). This was not observed in SENN females, where multiple meals did not result in a significant increase in enzyme activity (1 way ANOVA: p = 0.95, F = 0.8, df = 2) (Fig 6B).

## Discussion

Despite the potential importance of oxidative stress, it remains relatively unexamined in insects outside of the field of phytophagous insects. In this study, several observations can be made about how oxidative stress interacts with the insecticide resistance phenotype and longevity. This study demonstrates a variable response to oxidative stress in insecticide resistant and susceptible strains. This is highlighted by the fact that SENN DDT adults live longer under conditions of copper sulphate exposure and that insecticide selected individuals have an increased capacity to withstand hydrogen peroxide. These results are similar to the findings of Oliver and Brooke [18], which shows differential responses of the SENN and SENN DDT strains to multiple bloodmeals.

Furthermore, these differing responses appear to be related to the nature of the resistance mechanisms involved. Although the insecticide resistance phenotype in the SENN DDT strain is mediated by both target site and metabolic resistance, it appears that the initial primary resistance mechanism was the L1014F mutation [18,33]. In this strain, elevated capacity for coping with hydrogen peroxide is only observed in SENN DDT females. In contrast, in the FUMOZ-R strain, both males and females have an increased defence against hydrogen peroxide in comparison to the unselected FUMOZ strain. The primary resistance mechanism in the FUMOZ-R strain is increased metabolic detoxification due to increased production of the enzyme CYP6P9 [34] which has an increased affinity for pyrethroids [35]. The increased capacity for coping with oxidative stress in this strain may be have evolved to cope with the increased oxidative burden induced by increased Cytochrome P450 activity [36,37]. As such, it is possible to hypothesize that oxidative stress defence capacity is driven by the need to cope with the inherent burden induced by defence against insecticides [15].

All the evidence presented thus far has presented indirect evidence of the role of oxidative stress enzymes in insecticide resistance. Enzyme synergism provided a direct demonstration of the crucial role of oxidative stress enzymes in insecticide-induced toxicity. The direct role of oxidative stress enzymes in the resistance phenotypes assessed is demonstrated by the synergistic effects of superoxide dismutases, Glutathione S-transferases and catalase. Synergism was most effective in the FUMOZ-R strain, in which resistance is primarily mediated by P450 based metabolism, as opposed to the SENN DDT strain in which resistance is mediated by metabolic and target site resistance mechanisms. The most effective synergist was ATZ, a catalase synergist. This may be due to the limited number and distribution of catalases observed in *Anopheles* mosquitoes [38] which may simplify the synergism process. These findings suggest that the toxic effects of insecticide-induced oxidative stress play as crucial a role in toxicity as the neurotoxic effects of pyrethroids.

The hypothesis that increased defence against oxidative stress is associated with resistance to insecticides contrasts with a recent study on oxidative stress and longevity in *An*. *gambiae*. Decreased longevity in the RSP strain was attributed to an increase in quantifiable ROS, specifically H_2_O_2_ [13]. Similarly, longevity studies performed by Oliver and Brooke [18] also found a decrease in longevity in the insecticide resistant SENN DDT strain, but in this study it was also found that insecticide resistance associated with a greater defence against oxidative stress. This is in contrast to the findings of Otali *et al*. [13], which suggest that insecticide resistant *An*. *gambiae* has higher levels of mitochondrial ROS. The suggestion offered by our study is that the decreased longevity in the resistant strain may be due to a *kdr*-induced fitness cost [39].

The data presented here also give a possible explanation of how multiple bloodmeals augment and sustain the insecticide resistance phenotype in *An*. *arabiensis*. The question of how blood-feeding reduces insecticide toxicity is not a new question. An earlier study demonstrated that a decrease in post-bloodmeal toxicity was not related to cuticular penetration, insecticide metabolism or distribution [40]. This study suggests that modulation of the oxidative stress response underlies the augmentation of the insecticide resistance phenotype due to multiple blood-feeding in the ageing mosquito. This can be related to the increased GST activity observed in SENN DDT that had imbibed multiple bloodmeals [14]. This increased Glutathione peroxidase activity may be the cause of the decreased oxidative burden (protein carbonyl content) observed in SENN DDT that had taken multiple blood meals, regardless of whether the individuals were exposed to insecticides or not.

The study also gives a possible explanation as to why a single bloodmeal increases permethrin tolerance in pyrethroid resistant *An*. *funestus*. As resistant individuals have a higher basal defence against oxidative stress, the increased defence required to cope with ingested blood results in an increase in oxidative defence enzyme activity that, in turn, results in reduced pyrethroid toxicity. This may explain why a single blood meal reduces permethrin toxicity in resistant, but not susceptible, *An*. *funestus* [41].

It is concluded that defence against oxidative stress plays a role in the pyrethroid resistance phenotype in pyrethroid resistant *An*. *arabiensis* and *An*. *funestus* because pyrethroid intoxication induces oxidative damage in addition to its primary mode of action. This defence is mediated by both catalase and Glutathione peroxidase activity. Defence against oxidative damage is also strongly modulated by blood-feeding and inadvertently leads to reduced pyrethroid toxicity in pyrethroid resistant mosquitoes.
